# Postpartum Screening for Type 2 Diabetes Mellitus Among Women With Gestational Diabetes Mellitus at King Abdulaziz University Hospital: A Cross-Sectional Study

**DOI:** 10.7759/cureus.44273

**Published:** 2023-08-28

**Authors:** Suha Arab, Saleh Binmahfooz, Renad M Abualsaud, Alaa A Basuliman, Roba Qurain, Rawan H AlSaidlani, Shaker Alsharif, Maha Alsaiari, Hadeel Awami

**Affiliations:** 1 Department of Obstetrics and Gynecology, King Abdulaziz University Hospital, Jeddah, SAU; 2 Department of Medicine, King Abdulaziz University, Jeddah, SAU

**Keywords:** six weeks, saudi arabia, diabetes screening, postpartum, gestational diabetes mellitus (gdm)

## Abstract

Background

Diabetes is characterized by elevated blood glucose levels due to inadequate insulin production or abnormalities in cellular activity. Gestational diabetes mellitus (GDM) is one of the most prominent indicators of type 2 diabetes mellitus (T2DM), which develops in pregnant women whose pancreatic function is insufficient to control the insulin resistance associated with pregnancy. Moreover, it is the most common metabolic disorder, with the majority of cases beginning in the second or third trimester of pregnancy and affecting up to 25% of pregnant women.

Objectives

The objective of this study was to identify factors associated with postpartum T2DM screening in women with GDM at King Abdulaziz University Hospital (KAUH) between 2010 and 2022. The secondary objective was to assess the factors associated with providing information to the patients about the risks of increased blood glucose and postpartum lifestyle modification.

Methods

We conducted a retrospective cross-sectional study at KAUH to investigate potential factors associated with postpartum screening for T2DM. Out of 564 patients diagnosed with GDM between 2020 and 2022, we included 200 women aged over 18 years with a history of GDM, as they met the inclusion criteria for our study. Patients younger than 18 years with missing or incomplete baseline characteristics were excluded. Data were analyzed using SPSS Statistics version 21 (IBM Corp. Released 2012. IBM SPSS Statistics for Windows, Version 21.0. Armonk, NY: IBM Corp.), and p-value <0.05 was considered significant.

Results

A total of 200 postpartum women with GDM were included in this study. Their mean age was 35.02±5.2 years. Many of them had a family history of diabetes (83.0%) and a previous diagnosis of GDM (60.5%). The patients who performed glucose testing six weeks after birth were previously diagnosed with GDM (37.0%) or with a family history of diabetes (45.5%). The significant variables in this analysis were mothers having frequent postpartum hospital follow-up visits (P<0.001), mothers with gestational weight gain (P=0.018), those who were informed about the risks of increased blood glucose (P=0.011), and those who were informed about plans for postpartum glucose screening (P=0.002). The mothers with a previous history of GDM were the highest to be informed of the risks of elevated blood glucose.

Conclusion

Frequent postpartum hospital follow-up visits, gestational weight gain, knowledge of the risks of elevated blood sugar, and postpartum glucose screening plans were all associated with postpartum glucose testing rates among women with GDM in Saudi Arabia.

## Introduction

Diabetes is one of the most prevalent metabolic diseases that exists today. As morbidity and mortality rates increase, diabetes has surpassed cancer and cardiovascular disease to become the third "silent killer" in the world [1]. Over 425 million people worldwide were diagnosed with diabetes in 2017, with the number expected to rise to 629 million by 2045 (WHO, 2006). It is currently predicted that one in every three persons in the United States will have diabetes by 2025 [2]. In addition, there are three subtypes of diabetes, type 1, type 2, and gestational diabetes mellitus (GDM) [1] In particular, GDM is one of the key predictors of type 2 diabetes mellitus (T2DM), which occurs in pregnant women whose pancreatic function is inadequate to control the insulin resistance associated with pregnancy [3]. GDM is the most prevalent metabolic disorder, onset or initial diagnosis occurring in the second or third trimester of pregnancy, and it can impact up to 25% of pregnant women. A prior diagnosis of GDM is a recognized risk factor for later-life development of T2DM which underlines the significance of postpartum screening to identify individuals who are more likely to proceed and introduce disease prevention interventions [4]. Therefore, as with various diagnostic standards, the prevalence of GDM varies significantly. The International Association of Diabetes and Pregnancy Study Groups (IADPSG) criteria are the most widely used screening method internationally. According to the most current meta-analysis by Saeedi et al. (2021), an overview of the global prevalence of GDM was 14.7% worldwide [3]. Compared to women who have normoglycemic pregnancies, women with GDM have a sevenfold increased chance of developing T2DM. Therefore, early implementation of preventative treatments, such as lifestyle modifications and/or metformin medication, is advised for high-risk women. To detect persistent impaired glucose regulation, postpartum screening is necessary. Therefore, the current agreement advises starting an oral glucose tolerance test (OGTT) the fourth week following delivery and repeating the test every three years if normal glucose tolerance is found [5]. However, obesity, advanced maternal age, a family history of diabetes, and being overweight or obese are risk factors for GDM. The effects of GDM include a higher risk for cardiovascular disease in mothers, T2DM, macrosomia, and child delivery difficulties [6]. During the first diagnosis of GDM during the second or third trimester of pregnancy, timely detection of glucose tolerance postpartum is important because progression to T2DM can be reduced by about 40% by implementing lifestyle intervention programs after pregnancy [7]. The IADPSG/WHO criteria (fasting blood glucose 92 mg/dl and/or blood glucose 180 mg/dl at the first hour and/or 153 mg/dl at the second hour during an OGTT with 75 g of glucose) were used to determine the diagnosis of GDM, and based on the American Diabetes Association standards, the postpartum T2DM screening included a 75 g OGTT for measurement of fasting and two-hour blood glucose [5]. In general, the OGTT is recommended as preferential to the use of glycated hemoglobin (HbA1c, a blood glucose level) measure of the three-month average [4].

Furthermore, to the best of our knowledge and on reviewing the current medical literature, there is no data available on the prevalence and effect of screening in Saudi Arabia. Therefore, this study aims to assess the prevalence of postpartum screening for T2DM among women with GDM at King Abdulaziz University Hospital (KAUH).

## Materials and methods

Study design, setting, and time

We conducted a retrospective cross-sectional study at KAUH to investigate potential factors related to postpartum screening for T2DM. Of the 525 people diagnosed with GDM between 2020 and 2022, we enrolled all women over the age of 18 with a history of GDM in the study. We excluded women under 18 years of age and those who had diabetes before or after pregnancy. Three hundred and twenty-five patients either refused to participate, could not be reached by telephone, or had missing or incomplete baseline data and were, therefore, excluded from the study.

Ethical approval

The Unit of Biomedical Ethics at King Abdulaziz University Hospital granted ethical approval with reference No 99-23.

Data collection

To ensure accurate results, we created a questionnaire using Google Forms (Google LLC, Mountain View, California, USA), which we used to contact patients by phone. We followed the ethical protocol established by the university's committee for cross-sectional studies and obtained informed consent from the patients. Our questionnaire covered patients' sociodemographic details, family history of diabetes, chronic illnesses, and previous GDM diagnoses. We also inquired about their baseline glycated hemoglobin (HbA1c), pregnancy treatment, gestational weight gain, postpartum hospital follow-up visits, and whether they underwent a postpartum OGTT six weeks after delivery. Additionally, we asked about their mode of delivery and whether their newborn required admission to the neonatal intensive care unit (NICU).

Data entry and statistical analysis

We entered data through the Google questionnaire we created and then transferred it to Excel 16.70 (Microsoft Corp., Redmond, WA, USA) for data cleaning. Afterward, we analyzed the data using SPSS Statistics version 21 (IBM Corp. Released 2012. IBM SPSS Statistics for Windows, Version 21.0. Armonk, NY: IBM Corp.). We used numbers and percentages to categorize patients based on their sociodemographic and clinical characteristics. Furthermore, we used simple logistic regression analysis to identify factors associated with six weeks of postpartum glucose testing, informing patients about the risks of increased blood glucose, and postpartum lifestyle modification. A p-value equal to or less than 0.05 was considered statistically significant.

## Results

A total of 200 postpartum women with GDM were included in this study. Their age ranges from 23 to 47 years with a mean (±SD) of 35.02±5.2 years. The majority of the subjects are 33-38 years old (n = 84; 42.0%), have high school and above education (n = 165; 82.5%), and unemployed (n=132; 66.0%). Most get a monthly family income of 5000 to 10000 SAR (n = 91; 45.5%). Many of them are multiparous (n =172; 86.0%), with a family history of diabetes (n =166; 83.0%) and previous diagnosis of GDM (n =121; 60.5%). The majority of the subjects are without any underlying medical illness (n =138; 89.0%), and the majority of the mothers with illnesses have hypothyroidism (n =23; 11.5%). The first antenatal visit is before the 12th week of gestational age in most of them (n =174; 87.0%). The weight of the examined mothers changed with pregnancy. The majority of the subjects are 6-10 Kg (n =64; 32.0%). Most have high HbA1c, more than 6.5 mmol/L (n =93; 46.5%), and receive insulin with diet and physical activity (n =88; 44.0%). The delivery of many of them is by cesarean section (n =110; 55.0%), and the infant birth weight is 2500-3500 g in most of them (n =140; 70.0%). Table 1 shows the sociodemographic and clinical characteristics of the subjects.

**Table 1:** Sociodemographic and clinical characteristics of women with GDM (n=200) SR: Saudi riyal, GDM: gestational diabetes mellitus, HbA1c: hemoglobin A1c, SLE: systemic lupus erythematosus, T2DM: type 2 diabetes mellitus, T1DM: type 1 diabetes mellitus, SCA: spinocerebellar ataxia

					n	%
Age in years	Less than 27				10	5.0
	27-32				52	26.0
	33-38				84	42.0
	39-44				50	25.0
	More than 44				4	2.0
Educational level	Junior school and below				35	17.5
	High school and above				165	82.5
Occupation	Unemployed				132	66.0
	Employed				68	34.0
The family income per month in SR	Less than 5000 SR/M				44	22.0
	5000-10000 SR/M				91	45.5
	More than 10000 SR/M				65	32.5
Parity	Primiparous				28	14.0
	Multiparous				172	86.0
Family history of diabetes	No				34	17.0
	Yes				166	83.0
Previous diagnosis of GDM	No				79	39.5
	Yes				121	60.5
Any underlying medical illness		n	%	Yes	62	31.0
	No	138	69.0			
				SLE	1	.5
				Hepatitis B	1	.5
				Epilepsy	2	1.0
				Hypertension	8	4.0
				Hypothyroidism	23	11.5
				Asthma	3	1.5
				T2DM	15	7.5
				T1DM	6	3.0
				Glaucoma	1	.5
				SCA	2	1.0
Gestational age (in weeks) at the first antenatal visit to any clinic	Before 12^th^ week				174	87.0
	12^th^ week or after				26	13.0
Gestational weight change	Doesn't remember				48	24.0
	No change in weight				14	7.0
	Up to 5 kg gain				31	16.0
	6-10 kg gain				64	32.0
	11-15 kg gain				25	12.5
	16-20 kg gain				8	4.0
	More than 20 kg gain				8	4.0
	Lost weight				1	.5
Treatment for GDM during pregnancy	No treatment				10	5.0
	Metformin				30	15.0
	Diet and physical activity				72	36.0
	Insulin, diet, and physical activity				88	44.0
Glycated hemoglobin (HbA1c)	Doesn't remember				64	32.0
	Normal (6.5 mmol/L or less)				43	21.5
	High (more than 6.5 mmol/L)				93	46.5
Mode of delivery	Vaginal delivery				90	45.0
	Cesarean section				110	55.0
Infant birth weight in grams	Less than 2500 g				29	14.5
	2500-3500 g				140	70.0
	More than 3500 g				25	12.5
	Doesn't remember				6	3.0

The number of women who showed up in primary healthcare clinics for glucose testing at six weeks postpartum was 112 (56.0%). Moreover, 88 (43.5%) did not show up or did not remember during their appointments, which their attending healthcare providers set during their routine follow-up within the immediate postpartum period. The mothers with less education in this study did not perform glucose testing (OR=1.006; 95% CI=0.478-2.115; p=0.567) more than those with higher education. Likewise, unemployed mothers didn’t perform glucose testing (OR=1.305; 95% CI=0.72-2.363; p=0.234) more than employed ones. Besides, mothers in families with a monthly income of 10,000 SR or less didn’t perform glucose testing (OR=1.4; 95% CI=0.766-2.56; p=0.173) more than those with higher income. Moreover, primiparous mothers didn’t perform glucose testing (OR=1.712; 95% CI=0.757-3.876; p=0.138) more than multiparous. In addition, mothers with no previous diagnosis of GDM didn’t perform glucose testing (OR=1.623; 95% CI=0.914-2.88; p=0.065) more than those with a previous history of GDM. Furthermore, mothers with no weight gain or who didn't remember their weight change didn’t perform glucose testing (OR=1.981; 95% CI=1.088-3.608; p=0.018) more than those with weight gain. Moreover, mothers who didn’t receive treatment for GDM didn’t perform glucose testing (OR=1.627; 95% CI=0.424-6.246; p=0.352) more than those with weight gain. Likewise, mothers with normal or didn't remember their glycated hemoglobin didn’t perform glucose testing (OR=1.212; 95% CI=0.692-2.123; p=0.298) more than those with high glycated hemoglobin. Further, Mothers who delivered infants with weights up to 3500 g didn’t perform glucose testing (OR=1.206; 95% CI=0.514-2.833; p=0.417) more than those who delivered infants with weights over 3500 g. In addition, mothers who were not informed about the risks of increased blood sugar didn’t perform glucose testing (OR=2.381; 95% CI=1.178-4.811; p=0.011) more than those who were informed. Furthermore, mothers who were not informed about the postpartum glucose screening plans didn’t perform glucose testing (OR=3.623; 95% CI=1.976-6.642; p=0.002) more than those who were informed.

On the other hand, mothers with no family history of diabetes didn’t perform glucose testing (OR=0.684; 95% CI=0.316-1.481; p=0.220) less than those with a family history of diabetes. Likewise, mothers whose first antenatal care was before the 12th week didn’t perform glucose testing (OR=0.905; 95% CI=0.396-2.069; p=0.487) less than those whose first antenatal care was at the 12th week or after. Moreover, mothers who delivered vaginally didn’t perform glucose testing (OR=0.837; 95% CI=0.477-1.47; p=0.317) less than those who delivered with cesarean section. Furthermore, mothers whose infants weren't admitted to the NICU didn’t perform glucose testing (OR=0.847; 95% CI=0.447-1.604; p=0.364) less than those admitted to the NICU (Table 2).

**Table 2:** Factors associated with six weeks of postpartum glucose testing using simple logistic regression analysis (n=200) OR: odds ratio, CI: confidence interval, NICU: neonatal intensive care unit, GDM: gestational diabetes mellitus, HbA1c: hemoglobin A1C *p-value <0.05 is significant

Variable		Not performed glucose testing or don’t remember N= 88 (44%)	Performed glucose testing N= 112 (56%)	OR (95% CI)	p-value
Educational level	Junior school and below	15 (7.5%)	19 (9.5%)	1.006 (0.478-2.115)	0.567
	High school and above	73 (36.5%)	93 (46.5%)		
Occupation	Unemployed	61 (30.5%)	71 (35.5%)	1.305 (0.72-2.363)	0.234
	Employed	27 (13.5%)	41 (20.5%)		
Family income per month	10,000 SR or less	63 (31.5%)	72 (36.0%)	1.4 (0.766-2.56)	0.173
	More than 10,000 SR	25 (12.5%)	40 (20.0%)		
Parity	Primiparous	15 (7.5%)	12 (6.0%)	1.712 (0.757-3.876)	0.138
	Multiparous	73 (36.5%)	100 (50.0%)		
Family history of diabetes	No	12 (6.0%)	21 (10.5%)	0.684 (0.316-1.481)	0.220
	Yes	76 (38.0%)	91 (45.5%)		
Previous diagnosis of GDM	No	40 (20.0%)	38 (19.0%)	1.623 (0.914-2.88)	0.065
	Yes	48 (24.0%)	74 (37.0%)		
Any underlying medical illness	No	58 (29.0%)	79 (39.5%)	0.808 (0.443-1.471)	0.292
	Yes	30 (15.0%)	33 (16.5%)		
Gestational age at the first antenatal visit to any clinic, weeks	Before 12th week	76 (38.0%)	98 (49.0%)	0.905 (0.396- 2.069)	0.487
	12th week or after	12 (6.0%)	14 (7.0%)		
Gestational weight change	Don't remember or lost weight or zero gain	36 (18.0%)	29 (14.5%)	1.981 (1.088-3.608)	0.018*
	Weight gain	52 (26.0%)	83 (41.5%)		
Treatment for GDM during pregnancy	None	5 (2.5%)	4 (2.0%)	1.627 (0.424-6.246)	0.352
	Receives treatment or change in lifestyle	83 (41.5%)	108 (54.0%)		
Glycated hemoglobin (HbA1c)	Don't remember or normal (= or <6.5 mmol/L)	49 (24.5%)	57 (28.5%)	1.212 (0.692-2.123)	0.298
	Abnormal (>6.5 mmol/L)	39 (19.5%)	55 (27.5%)		
Mode of delivery	Vaginal delivery	37 (18.5%)	52 (26.0%)	0.837 (0.477-1.47)	0.317
	Cesarean section	51 (25.5%)	60 (30.0%)		
Infant birth weight	Don't remember or weigh up to 3500 g	78 (39.0%)	97 (48.5%)	1.206 (0.514-2.833)	0.417
	Weight is >3500 g	10 (5.0%)	15 (7.5%)		
NICU admission	No	64 (32.0%)	85 (42.5%)	0.847 (0.447-1.604)	0.364
	Yes	24 (12.0%)	27 (13.5%)		
Postpartum hospital follow-up visits	No	55 (27.5%)	21 (10.5%)	7.222 (3.803-13.716)	<0.001*
	Yes	33 (16.5%)	91 (45.5%)		
Healthcare providers informed about the risks of your increased blood glucose	No or don’t remember	25 (12.5%)	16 (8.0%)	2.381 (1.178-4.811)	0.011*
	Yes	63 (31.5%)	96 (48.0%)		
Healthcare providers informed about the postpartum glucose screening plans	No or don’t remember	46 (23.0%)	26 (13.0%)	3.623 (1.976-6.642)	0.002*
	Yes	42 (21.0%)	86 (43.0%)		

It was discovered that mothers aged 33-38 years are the most likely to be informed about the risks of elevated blood glucose (n =55; 27.5%) and postpartum lifestyle modification (n =68; 34.0%). The same goes for unemployed mothers (n =103; 66.5% and n =92; 46.0%) and high school graduates and above (n =133; 66.5% and n =117; 58.5%). Many of the informed mothers are multiparous (n =139; 69.5% and n =123; 61.5%), with a family monthly income of 5000-10000 SR (n =73; 36.5% and n =64; 32.0%) and with a family history of diabetes (n =139; 68.5% and n =123; 61.5%). The highest number of the informed mothers are with a previous history of GDM (n =99; 49.5% and n =90; 45.0%) but without any underlying medical illness (n =110; 55.0% and n =101; 50.5%). The majority of the informed mothers were before the 12th week of gestation (n =139; 69.5% and n =121; 60.5%) who gained 6-10 kg in pregnancy (n =49; 24.5% and n =45; 22.5%) and received insulin in treatment (n =70; 35.0% and n =66; 33.0%). Most of the informed mothers have high HbA1c (n =79; 39.5% and n =71; 35.5%), delivered through cesarean section (n =88; 44.0% and n =77; 38.5%), and with infants that weighed 2500-3500 gm (n =118; 59.0% and n =100; 50.0%). Many of the informed mothers were under postpartum hospital follow-up (n =100; 50.0% and n =87; 43.5%) but with no NICU admission (n =120; 60.0% and n =102; 51.0%) (Table 3).

**Table 3:** Factors associated with information given to the patients about the risks of increased blood glucose and postpartum lifestyle modification (n=200) SR: Saudi rial, GDM: gestational diabetes mellitus, HbA1c: hemoglobin A1c, NICU: neonatal intensive care uni, SLE: systemic lupus erythematosus, T2DM: type 2 diabetes mellitus, T1DM: type 1 diabetes mellitus, SCA: spinocerebellar ataxia P-value <0.05 is significant

		Healthcare providers informed the patient about the risks of increased blood glucose					Healthcare providers informed about postpartum lifestyle modification				
		No		Yes		p-value	No		Yes		p-value
		n	%	n	%		n	%	n	%	
Age in years	Less than 27	6	(3.0%)	3	(1.5%)	0.464	3	(1.5%)	6	(3.0%)	0.514
	27-32	18	(9.0%)	35	(17.5%)		19	(9.5%)	33	(16.5%)	
	33-38	26	(13.0%)	55	(27.5%)		16	(8.0%)	68	(34.0%)	
	39-44	13	(6.5%)	37	(28.5%)		16	(8.0%)	34	(17.5%)	
	More than 44	1	(0.5%)	3	(1.5%)		2	(1.0%)	2	(1.0%)	
Educational level	Junior school and below	9	(4.5%)	26	(13.0%)	0.007	11	(5.5%)	24	(12.0%)	0.071
	High school and above	32	(16.0%)	133	(66.5%)		48	(24.0%)	117	(58.5%)	
Occupation	Unemployed	28	(14.0%)	103	(51.5%)	0.583	39	(19.5%)	92	(46.0%)	0.998
	Employed	13	(6.5%)	55	(27.5%)		20	(10.0%)	48	(24.0%)	
Family income per month in SR	Less than 5000 SR/M	12	(6.0%)	32	(16.0%)	0.459	16	(8.0%)	28	(14.0%)	0.289
	5000-10000 SR/M	18	(9.0%)	73	(36.5%)		27	(13.5%)	64	(32.0%)	
	More than 10000 SR/M	11	(5.5%)	54	(27.0%)		16	(8.0%)	49	(24.5%)	
Parity	Primiparous	8	(4.0%)	20	(10.0%)	0.327	10	(5.0%)	18	(9.0%)	0.451
	Multiparous	33	(16.5%)	139	(69.5%)		49	(24.5%)	123	(61.55)	
Family history of diabetes	No	10	(5.0%)	24	(12.0%)	0.204	5	(2.5%)	29	(14.5%)	0.126
	Yes	31	(15.5%)	135	(67.5%)		54	(27.0%)	112	(56.0%)	
Previous diagnosis of GDM	No	19	(9.5%)	60	(30.0%)	0.162	28	(14.0%)	51	(25.5%)	0.48
	Yes	22	(11.0%)	99	(49.5%)		31	(15.5%)	90	(45.0%)	
Any underlying medical illness	No	28	(14.0%)	110	(55.0%)	0.542	37	(18.5%)	101	(50.5%)	0.593
	Yes	13	(6.5%)	49	(24.5%)		22	(11.0%)	40	(20.0%)	
	SLE	1	(0.5%)	0	(0.0%)		1	(0.5%)	0	(0.0%)	
	Hepatitis B	0	(0.0%)	1	(0.5%)		1	(0.5%)	0	(0.0%)	
	Epilepsy	0	(0.0%)	2	(1.0%)		1	(0.5%)	1	(0.5%)	
	Hypertension	3	(1.5%)	5	(2.5%)		4	(2.0%)	4	(2.0%)	
	Hypothyroidism	4	(2.0%)	19	(9.5%)		7	(3.5%)	16	(8.0%)	
	Asthma	0	(0.0%)	3	(1.5%)		1	(0.5%)	2	(1.0%)	
	T2DM	3	(1.5%)	12	(6.0%)		4	(2.0%)	11	(5.5%)	
	T1DM	1	(0.5%)	5	(2.5%)		3	(1.5%)	3	(1.5%)	
	Glaucoma	0	(0.0%)	1	(0.5%)		0	(0.0%)	1	(0.5%)	
	SCA	1	(0.5%)	1	(0.5%)		0	(0.0%)	2	(1.0%)	
Gestational age (in weeks) at first antenatal visit	Before 12^th^ week	35	(17.5%)	139	(69.5%)	0.297	53 (26.5%)		121	(60.5%)	0.348
	12^th^ week or after	6	(3.0%)	20	(10.0%)		6 (3.0%)		20	(10.0%)	
Gestational weight change	Doesn't remember	10	(5.0%)	38	(19.0%)	0.943	16	(8.0%)	32	(16.0%)	0.480
	No change in weight	1	(0.5%)	13	(6.5%)		3	(1.5%)	11	(5.5%)	
	Up to 5 kg gain	6	(3.0%)	26	(13.0%)		7	(3.5%)	25	(12.5%)	
	6-10 kg gain	15	(7.5%)	49	(24.5%)		19	(9.5%)	45	(22.5%)	
	11-15 kg gain	5	(2.5%)	20	(10.0%)		10	(5.0%)	15	(7.5%)	
	16-20 kg gain	1	(0.5%)	7	(3.5%)		2	(1.0%)	6	(3.0%)	
	More than 20 kg gain	3	(1.5%)	5	(2.5%)		1	(0.5%)	7	(3.5%)	
	Lost weight	0	(0.0%)	1	(0.5%)		1	(0.5%)	0	(0.0%)	
Treatment for GDM during pregnancy	No treatment	5	(2.5%)	4	(2.0%)	0.038	5	(2.5%)	4	(2.0%)	0.360
	Metformin	1	(0.5%)	29	(14.5%)		7	(3.5%)	23	(11.5%)	
	Diet and physical activity	17	(8.5%)	56	(28.0%)		25	(12.5%)	48	(24.0%)	
	Insulin, diet, and physical activity	18	(9.0%)	70	(35.0%)		22	(11.0%)	66	(33.0%)	
Glycated hemoglobin (HbA1c)	Doesn't remember	17	(8.5%)	46	(23.0%)	0.142	22	(11.0%)	41	(20.5%)	0.306
	Normal (6.5 mmol/L or less)	10	(5.0%)	34	(17.0%)		15	(7.5%)	29	(14.5%)	
	High (more than 6.5 mmol/L)	14	(7.0%)	79	(39.5%)		22	(11.0%)	71	(35.5%)	
Mode of delivery	Vaginal delivery	19	(9.5%)	71	(35.5%)	0.968	26	(13.0%)	64	(32.0%)	0.922
	Cesarean section	22	(11.0%)	88	(44.0%)		33	(16.5%)	77	(38.5%)	
Infant birth weight in grams	Less than 2500 g	8	(4.0%)	20	(10.0%)	0.002	10	(5.0%)	18	(9.0%)	0.071
	2500-3500 g	22	(11.0%)	118	(50.9%)		40	(20.0%)	100	(50.0%)	
	More than 3500 g	8	(4.0%)	17	(8.5%)		6	(3.0%)	19	(9.5%)	
	Doesn't remember	3	(1.5%)	3	(1.5%)		3	(1.5%)	3	(1.5%)	
NICU admission	No	29	(14.5%)	120	(60.0%)	0.659	47	(23.5%)	102	(51.0%)	0.470
	Yes	12	(6.0%)	39	(19.5%)		12	(6.0%)	39	(19.5%)	
Postpartum hospital follow-up visits	No	17	(8.5%)	58	(29.0%)	0.828	35	(17.5%)	40	(20.0%)	0.039
	Yes	24	(12.0%)	100	(50.0%)		37	(18.5%)	87	(43.5%)	

## Discussion

The results of this study shed light on how frequently postpartum glucose testing is performed among Saudi Arabian women with GDM. The study discovered that almost 56.0% of the participants visited primary healthcare clinics for glucose testing six weeks after giving birth, showing a comparatively higher adherence to postpartum screening compared to a prior study six weeks after giving birth compared to a prior study carried out in China [8]. In addition, previous research done in Malaysia to determine the proportion of the percentage of women diagnosed with GDM who had a postpartum glucose test, as well as the variables influencing test compliance, found that six weeks after giving birth, 35.8% of 341 mothers visited primary healthcare clinics for glucose testing [9]. This increased adherence in Saudi Arabian citizens shows that they are more aware of and cognizant of the significance of postpartum diabetes screening.

It is essential to pinpoint the causes of greater postpartum glucose testing rates in order to create focused treatments and raise screening rates. According to the results of our study, having a more significant change in gestational weight, knowledge of the risks of elevated blood sugar, postpartum glucose screening plans, and frequent postpartum hospital follow-up visits were all significantly associated with increased adherence to postpartum glucose testing. These factors are likely to be related to increased adherence to follow-up and screening, as the mother has already received injectable treatments, which many patients don't prefer, and a higher HbA1c puts her at a higher risk of complications, which she would have been informed about during her antenatal care. These results are consistent with other studies, which found that women tend to avoid screening programs due to a lack of education, a negative opinion of the screening modalities, and subpar laboratory conditions [10,11]. Other previous research done in Italy was obtained to determine what factors affected the likelihood of women with GDM attending early postpartum follow-up appointments and discovered that 741 (83.4%) of the 889 (48.9%) participants in the postpartum OGTT who showed up had normal glucose tolerance, whereas 148 (16.6%) had abnormal glucose tolerance (impaired fasting glucose (IFG) 6.7%, impaired glucose tolerance (IGT) 7.7%, IFG + IGT 0.8%, DM2 1.5%). Not being a member of an immigrant group, having a family history of diabetes, and using insulin during pregnancy were the predictors of follow-up adherence [12]. It emphasizes the significance of comprehensive healthcare and support systems that encourage and facilitate postpartum glucose testing while taking into account socioeconomic, clinical, and personal difficulties.

In this study, sociodemographic factors such as age, ethnicity, educational level, and employment status did not significantly influence postpartum glucose testing behavior. This also aligns with previous studies that stated them as independent factors and had no effect on the screening rate [13]. There is very scarce research investigating the factors affecting postpartum screening; therefore, we have yet to see opposing evidence that may contribute to cultural differences. This emphasizes the need for further research to explore the contextual factors that impact postpartum screening behavior in different populations.

The clinical importance of postpartum screening for T2DM in patients with GDM has previously been studied in Missouri. The study determined that only 9.7% were screened at 12 weeks, and only 20.8% were screened at 18 months, underscoring the need for health education and regular screening for T2DM [14]. Another systematic review and meta-analysis, including 20 studies and a total of 1,332,373 individuals, concluded that women with GDM have a tenfold risk of developing T2DM compared to healthy controls, further highlighting the need for screening to prevent the onset of T2DM, particularly in the early years [4]. Enhancing postpartum glucose testing rates among women with GDM remains crucial for detecting and effectively managing T2DM. Future interventions should address barriers to postpartum screening, particularly among women with lower family income, no family history of diabetes, and those who did not have regular postpartum hospital follow-up visits. Implementing targeted educational campaigns, providing accessible and affordable screening options, and improving communication between healthcare providers and patients can enhance postpartum screening rates. Improving screening rates and ensuring timely intervention can mitigate the long-term health risks associated with GDM, leading to better health outcomes for women and their offspring.

All studies have their strengths and weaknesses, and our study is not excluded. Our study has shed light on the factors that increase adherence to glucose testing postpartum that affect women living in the Mid-gulf, where, to our knowledge, no other studies have been conducted in this area. However, the study's cross-sectional nature may have limited our access to other detailed factors.

## Conclusions

This study in Saudi Arabia found that women with GDM are more likely to receive postpartum glucose testing if they have frequent postpartum hospital follow-up visits, knowledge of the risks of elevated blood sugar, postpartum glucose screening plans, and greater gestational weight change. Sociodemographic factors did not appear to affect postpartum diabetes screening in this study. Future research should investigate additional risk factors that may impact postpartum T2DM screening and focus on improving screening methods to make them more accessible and convenient for women with GDM.
